# The direct and indirect drivers shaping RNA viral communities in grassland soils

**DOI:** 10.1128/msystems.00099-24

**Published:** 2024-07-09

**Authors:** Ruonan Wu, Amy E. Zimmerman, Kirsten S. Hofmockel

**Affiliations:** 1 Earth and Biological Sciences Directorate, Pacific Northwest National Laboratory, Richland, Washington, USA; 2 Department of Agronomy, Iowa State University, Ames, Iowa, USA; Quadram Institute Bioscience, Norwich, Norfolk, United Kingdom

**Keywords:** soil RNA viruses, mycoviruses, water content, plant, soil depth

## Abstract

Recent studies have revealed diverse RNA viral communities in soils. Yet, how environmental factors influence soil RNA viruses remains largely unknown. Here, we recovered RNA viral communities from bulk metatranscriptomes sequenced from grassland soils managed for 5 years under multiple environmental conditions including water content, plant presence, cultivar type, and soil depth. More than half of the unique RNA viral contigs (64.6%) were assigned with putative hosts. About 74.7% of these classified RNA viral contigs are known as eukaryotic RNA viruses suggesting eukaryotic RNA viruses may outnumber prokaryotic RNA viruses by nearly three times in this grassland. Of the identified eukaryotic RNA viruses and the associated eukaryotic species, the most dominant taxa were *Mitoviridae* with an average relative abundance of 72.4%, and their natural hosts, Fungi with an average relative abundance of 56.6%. Network analysis and structural equation modeling support that soil water content, plant presence, and type of cultivar individually demonstrate a significant positive impact on eukaryotic RNA viral richness directly as well as indirectly on eukaryotic RNA viral abundance via influencing the co-existing eukaryotic members. A significant negative influence of soil depth on soil eukaryotic richness and abundance indirectly impacts soil eukaryotic RNA viral communities. These results provide new insights into the collective influence of multiple environmental and community factors that shape soil RNA viral communities and offer a structured perspective of how RNA virus diversity and ecology respond to environmental changes.

**IMPORTANCE:**

Climate change has been reshaping the soil environment as well as the residing microbiome. This study provides field-relevant information on how environmental and community factors collectively shape soil RNA communities and contribute to ecological understanding of RNA viral survival under various environmental conditions and virus-host interactions in soil. This knowledge is critical for predicting the viral responses to climate change and the potential emergence of biothreats.

## INTRODUCTION

Viruses represent a critical component of environmental microbiomes. This is because viral infection of microbial hosts can have cascading effects on ecosystem function by augmenting host metabolism/phenotype, population dynamics, community interactions, and host evolution (as gene transfer agents) (1, 2). Studies of environmental viruses have focused primarily on viruses with DNA genomes. This is partially due to the challenges of recovering sufficient RNA from water or soil for sequencing and detecting viral signals within a high background of ribosomal RNA sequences (3, 4). These challenges are exacerbated by the small genome size of many RNA viruses (5). Thus, the genomic diversity of RNA viruses remains substantially under-sampled across environments, with each new study extending the boundaries of the known RNA virosphere (6–10).

RNA viruses’ ecological and evolutionary impacts on environmental microbiomes are likely distinct from DNA viruses due to key differences in their fundamental biology. For example, a diversity of hosts are infected by RNA viruses (including bacteria, archaea, and microbial eukaryotes, as well as higher eukaryotes like plants and arthropods) (11), while studies of dsDNA viruses mainly capture bacteria-infecting viruses (bacteriophages or phages). Additionally, the diversity of eukaryotic hosts tends to be higher than prokaryotic hosts (12, 13), infections may be cryptic without obvious changes in host phenotype (14), and many RNA viruses are vertically transmitted without an extracellular state (15). Soils, in particular, appear to harbor a vast reservoir of RNA viral diversity (8, 10, 13), with a high prevalence of RNA bacteriophages in addition to eukaryotic RNA viruses (7, 9, 12, 16). Despite the potential for substantial and distinct impacts of RNA viruses on environmental microbiomes, our understanding of their diversity and ecology remains underexplored.

Distinct RNA viral communities have been observed across broad soil types (8, 10, 13), and investigating the specific factors shaping RNA viral communities in soil is an area of active study. Host community composition is an obvious driver of viral community composition, given the parasitic nature of viruses (17–19). Therefore, factors that influence soil bacteria, archaea, and eukaryotes, such as soil pH, soil water content, and other biotic and abiotic factors (20, 21), may indirectly shape the viral component of soil microbiomes. This is supported by evidence of viral responses to soil water content (16), though direct effects of moisture may also contribute. Plant and rhizosphere effects encompass a suite of interrelated factors that also influence the RNA viral community composition (9, 12, 22). Other evidence suggests that drivers of viral community composition may be decoupled from those of their hosts. For example, a significant distance-decay relationship was observed in phage communities detected in California grassland soils but not in the co-existing bacterial communities (23). Our current understanding of the potential factors shaping RNA viral communities in soils has been derived from the enrichment of extracellular virus particles (10), which excludes the substantial proportion of RNA viruses that are expected to be obligate intracellular viruses (14), and from a few microcosm-scale experiments, which substantially alter the environmental conditions of the microbiome prior to analysis (12, 13, 16). Thus, a key knowledge gap remains about the relative contributions of multiple potential drivers shaping the composition of soil RNA viral communities *in situ*.

Here, we address this knowledge gap by evaluating the direct and indirect effects of environmental factors including soil water content (water holding capacity or WHC), soil depth (depth), plant presence (planted and bare soils), and plant genotype (type of cultivar) on soil RNA viral communities. We hypothesize that environmental factors directly influence the composition and associated genetic diversity of soil RNA viral communities as well as indirectly through the potential host communities. The RNA viral communities and the co-existing prokaryotic and eukaryotic communities were recovered from the total RNA metatranscriptomes sequenced from soils with a range of field manipulations. The design of the field experiment includes high and low irrigation regimes (creating soils with different water contents) across planted and unplanted plots (plant presence) with two genotypes of perennial tall wheatgrass (*Thinopyrum ponticum*; cultivar type) (24), thus enabling us to evaluate the contributions of several environmental factors within the same field site. Our field site was planted in May 2018 at the Washington State University Irrigated Agriculture Research and Extension Center located in Prosser, WA, and represents an arid grassland ecosystem with marginal soil, as previously described (25). Grasslands are globally important ecosystems that provide a variety of ecosystem services and marginal grasslands may hold additional potential for bioenergy crop production and carbon sequestration (26). Irrigation treatments at our field site have been ongoing for 5 years (since spring 2019), allowing us to test environmental treatment effects on RNA viral communities. Field-relevant evidence from this study advances understanding of how environmental and community factors collectively influence the composition of soil RNA viruses and provides a conceptual framework for how virus-host relationships shift in response to environmental changes.

## MATERIALS AND METHODS

### Soil sampling and RNA extraction

**Fig 1:**
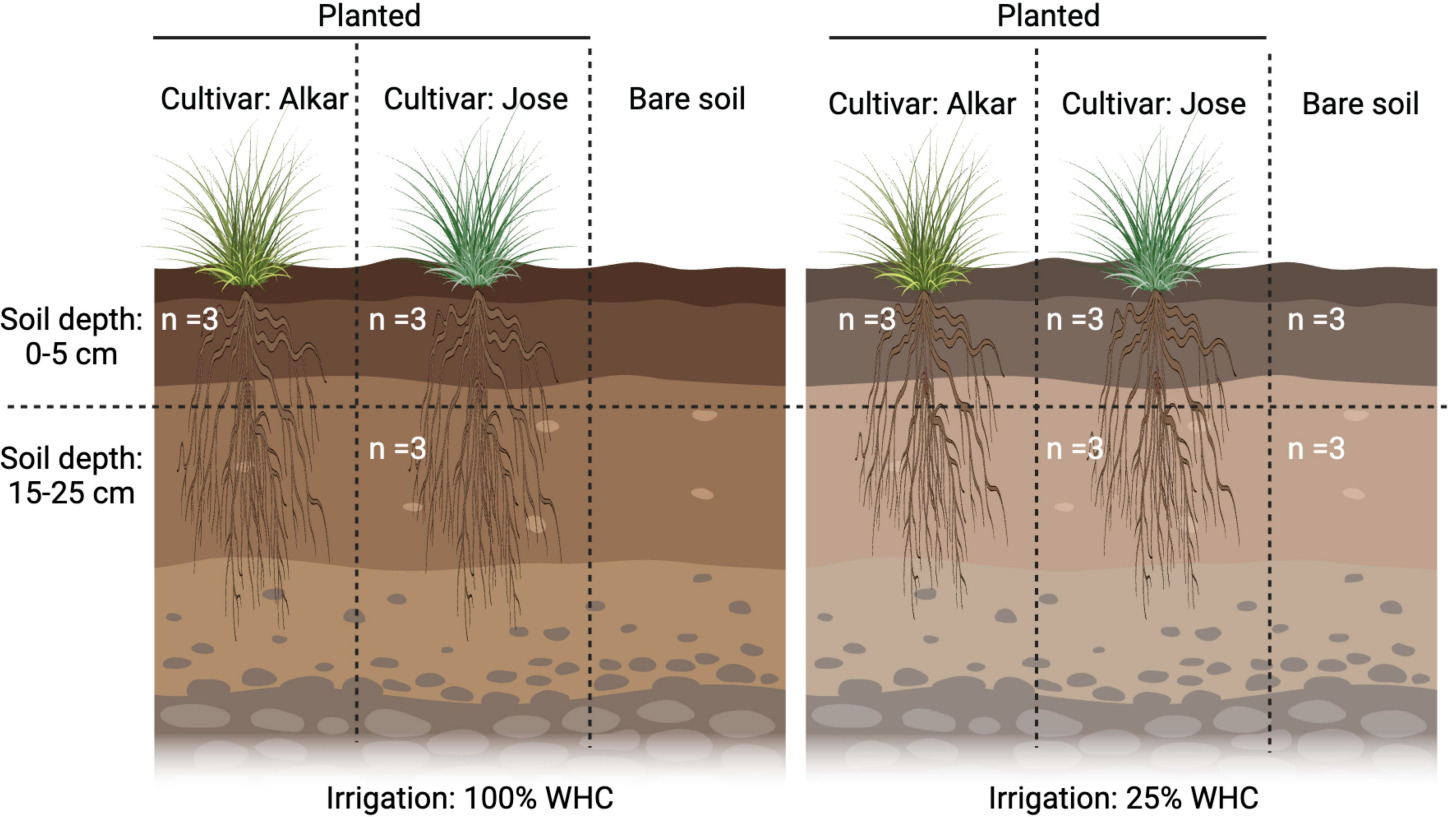
Schematic of the field experiment. Replicate soil plots within the same grassland field site were planted with two types of tall wheatgrass cultivars (cultivar: Alkar or Jose) and under two irrigation regimes (irrigation intensity: 100% or 25% of WHC). Each experimental plot is 2.1 m × 10.7 m with a 1.5 m alley between adjacent plots. Grasses with primary vertical roots fully occupy the planted plot. Within a plot, four independent soil cores were collected from random locations within 15.2 cm of the nearest plant. The soil plots without wheatgrass adjacent to the planted plots were also sampled (bare soil). Soil samples were collected from two depth profiles (soil depth: 0–5 cm or 15–25 cm). The number of replicate samples collected from each unique soil plot is indicated by “n.”

On 15 October 2020, bulk soil samples were randomly collected from 24 plots within the Tall Wheatgrass Irrigation Field Trial in Prosser WA, USA (46°15′04″N and 119°43′43″W) (25). Each experimental plot is 2.1 m × 10.7 m with a 1.5 m alley between adjacent plots. The sampled plots represent soils with different combinations of environmental treatments, each in three biological replicates (see Fig. 1 for experimental design). The 24 samples include three field replicates of surface (0–5 cm) soils planted with two cultivars (Jose and Alkar) at two irrigation levels (irrigation intensity: 25% and 100% of WHC) (*n* = 12); three field replicates of subsurface soil (15–25 cm) planted with Jose cultivar for both irrigation treatments (*n* = 6); and two soil depths for the bare plots (0–5 cm and 15–25 cm; *n* = 6). Within each plot, four independent cores (91.4 cm long, 1.9 cm diameter) were aseptically collected from random locations within 15.2 cm of the nearest plant. Tall wheatgrass has continuous coverage in each plot with primarily vertical roots. The four cores were composited to generate one representative sample per plot. Samples were transported to the Pacific Northwest National Laboratory (PNNL) on ice, flash-frozen in liquid N_2_, and stored at −80°C prior to RNA extraction. The soil water content of each sample was measured by the gravimetric method (24). More details about the design of the field experiment and soil sampling can be found in our previous publication (24).

Total RNA was extracted using the Zymo Quick-RNA fecal/soil microbe miniprep (Zymo Research, Irvine, CA), incorporating the DNase I treatment using Zymo’s DNase I kit (Zymo Research) by following the manufacturer’s instructions with modifications [i.e., doubling the amount of soil per extraction (from 0.25 g to 0.5 g) and pooling triplicated extractions as one]. RNA concentration was assessed using a Qubit RNA HS assay kit (Thermo Fisher, Waltham, MA), and RNA quality was determined using an Agilent 2100 BioAnalyzer (Agilent, Santa Clara, CA).

### Metatranscriptome sequencing and *de novo* assembly

The total RNA extracted from each soil sample was sequenced by GENEWIZ/Azenta (GENEWIZ/Azenta Life Sciences, South Plainfield, NJ) using an Illumina platform. The raw metatranscriptome data were deposited with our previous publication (24). The raw reads of the metatranscriptomes used in this study were trimmed and quality-filtered by Trimmomatic (v0.33) using the default parameters. The trimmed reads were further aligned to the PhiX genome by Burrows-Wheeler Aligner (v0.7.17). The exact matches were filtered out to remove the potential PhiX contamination commonly used as a control for Illumina sequencing. Ribosomal RNA reads were bioinformatically removed using SortMeRNA (v4.3.4). The remaining reads of each metatranscriptome were *de novo* assembled using MEGAHIT (v1.2.9) with the default parameters. The description of each metatranscriptome used in this study is included in Table S1.

### Identification of RNA viral sequences

The assembled contigs were subjected to RNA viral sequence identification via two methods, the presence of RNA-dependent RNA polymerase (RdRP) or the high sequence similarity to the RNA viral genomes in the reference databases curated in this study (12, 13, 16). Genes of the assembled contigs were predicted and translated using Prodigal (v2.6.3). The protein sequences were searched against a suite of RdRP Hidden Markov Models (HMMs) using hmmsearch (Hmmer v3.1b2) (13). Putative RdRP sequences were identified with cutoffs of coverage ≥ 50%, e-value < 1e−10, and score ≥ 70. The RdRP HMM search method was applied as the primary method to capture the sequence diversity in soil RNA viruses and complemented with a viral genome searching method using stringent cutoffs to minimize the detection of potential false positives. The RNA viral reference database was composed of 6,621 complete RNA viral genomes collected from the NCBI virus database (accessed on 12 August 2022), 378,253 RNA viral contigs from the RVMT database (v3) (7), and 858 RNA viral genomes published in a terrestrial RNA viral study (8). The assembled contigs were queried against the curated RNA viral reference database using BLASTN (v2.13.0) and screened at the cutoffs of e-value < 1e−10, percent of identity > 90%, and coverage > 50%. The RNA viral contigs that were not detected in the curated viral reference database using the searching criteria were considered novel.

### Taxonomic and host assignment of the RNA viral contigs

To avoid overestimation of the RNA viral diversity, RdRP, the phylogenetic marker of RNA viruses, was used for clustering the RNA viral contigs and making the taxonomic assignment as demonstrated previously (12, 16). For the RNA viral contigs that were lacking RdRP genes and thus identified via searching against the curated reference database, they inherited the RdRP gene from the most closely related RNA viral genome in the database (e-value < 1e−10, percent of identity > 90%, and coverage > 50%) and its taxonomic assignment. The RdRPs directly detected in identified RNA viral contigs were clustered with these assigned RdRPs at 99% of identity by CD-HIT (v 4.8.1). The number of RdRP clusters normalized by the sequencing depth of each sample was used to represent the richness of the detected RNA viral community. We acknowledge that RNA viruses without an RdRP (e.g., retroviruses) were not investigated in this study. Taxonomic and putative host assignments of the identified RNA viral contigs were made according to the classified RNA viral reference genomes in the same RdRP cluster. The average read coverage of the detected RNA viral contigs was calculated to estimate the relative abundance of the recovered RNA viruses. The quality-filtered forward reads were aligned to the identified RNA viral contigs and filtered at the percent of identity greater than 95% and coverage higher than 80% by BamM (v1.7.3, bamm make and bamm filter). We then used samtools (v1.9, samtools depth) to calculate the read coverage per base for each contig. The average count of the total quality-filtered forward reads of the 24 metatranscriptomes was calculated to normalize each of the contig coverage. The normalized contig coverage was then used to estimate the relative abundances of the identified RNA viral contigs and compared across samples.

### Detections of potentially active eukaryotes and prokaryotes

The small subunit (SSU) rRNAs were used to detect the co-existing eukaryotes and prokaryotes in each sample. The forward metatranscriptomic reads without rRNA removal were aligned to the SILVA prokaryotic and eukaryotic SSU databases (release 138.1, Ref NR 99) and filtered using the same method mentioned above. The transcript abundances of the prokaryotic and eukaryotic members were estimated by the average base coverage of the mapped SSU rRNA reference sequences normalized by the total counts of reads per sample.

### Statistical analyses

To compare the relative complexity of the five communities recovered across the 24 soil samples, principal component analysis on the composition of each community (RdRP- or SSU rRNA-informed abundance matrix) was performed using “prcomp” in R (v4.3.0), and the cumulative proportion of variance explained with an increasing number of principal components was calculated for each community type (i.e., eukaryotic community, eukaryotic RNA viral community, prokaryotic community, prokaryotic RNA viral community, and the total RNA viral community). The compositions between treatments were compared by calculating Bray-Curtis dissimilarity using the Function “vegdist” and the significance of the differences between each pair was evaluated by *t*-test. The nonmetric multidimensional scaling (NMDS) analysis was performed using the Function “metaMDS” in R (v4.3.0). The significance of the environmental factors’ impacts on community composition was assessed by permutational multivariate analysis of variance (PERMANOVA) using the Function “adonis2,” which performs an F-test with 999 permutations.

We performed Pearson correlations and random forest analyses to investigate the associations between the environmental factors (i.e., water content, presence of plant, type of cultivar, and soil depth) and soil biological community factors (i.e., abundance, richness, and the first principal component or PC1 of eukaryotic, prokaryotic, or RNA viral communities used to estimate beta diversity). Pearson correlations were computed and their significance was evaluated using the “Hmisc” package (*P* < 0.05). We further conducted random forest analysis, a machine learning method, to investigate the relative contribution of each environmental factor and, eukaryotic and prokaryotic community factor in predicting the eukaryotic/prokaryotic/total RNA viral abundance and richness using the “rfPermute” package with permutations for each model (ntree = 100, num.rep = 50). The significant predictors (*P* < 0.05) were evaluated based on the percentage increase in mean squared error (%IncMSE) and an increase in node purity (IncNodePurity), a representation of the relative variable importance. The cross-validated r^2^ and the *P*-value of each model were generated using the “A3” package. The correlation coefficients of the significant relationships detected in the correlation and random forest analyses were used to generate a factor network using a greedy modularity optimization algorithm to detect the closely connected factors within a specific module with fewer connections across modules using the “igraph” package.

We applied structural equation modeling (SEM) to test the hypothesis that the soil eukaryotic RNA viruses are strongly influenced by the eukaryotic members and respond to the environmental factors directly and indirectly. The model structure was based on the significant relationships detected by the correlation and random forest analysis and the module structure identified in the network analysis. A Chi-square (χ2) test was performed and the comparative fit index (CFI) was calculated to evaluate the model fit (cutoffs: *P*-value of the χ2 test > 0.05, CFI > 0.8). After selecting the model with the best fit to the data, the R package “semPaths” was used to create the SEM diagram with the result of path analysis. The connection and strength of the resolved paths in the SEM model were evaluated by path coefficients representing the change of dependent variable with a unit change in the explanatory variable and *P*-values demonstrating the significance of the paths (*P* < 0.05 was considered significant in this study). The direct impact of one variable on another that was supported by the SEM was plotted as a directional arrow.

## RESULTS

### The predominance of eukaryotic RNA viruses

**Fig 2:**
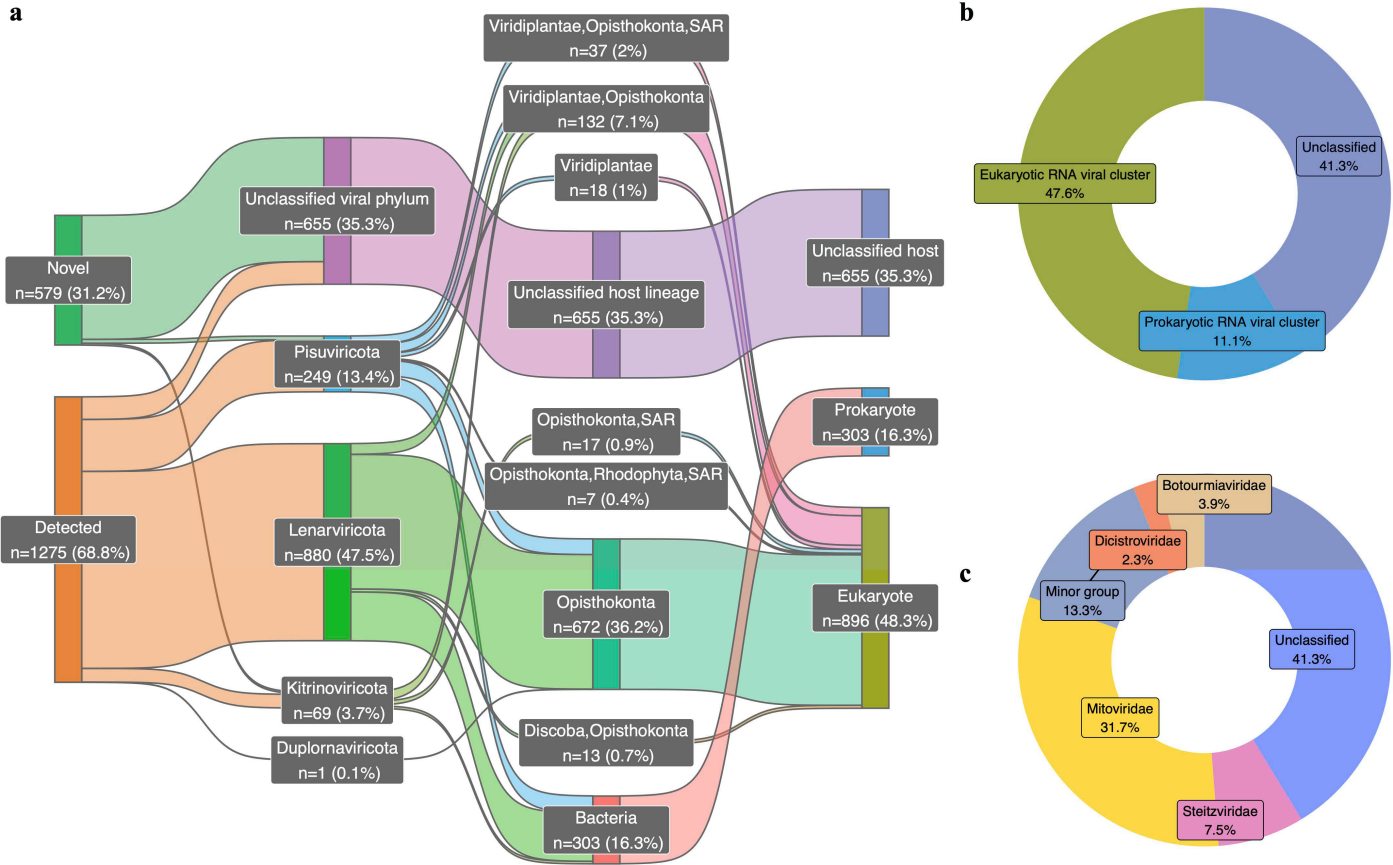
Taxonomic classification and the range of the known hosts of the RNA viruses identified in the studied grassland soils. (a) Taxonomic classification and the known hosts of the 1,854 RNA viral contigs. From left to right, a Sankey plot shows the exact numbers and the proportions of the RNA viral contigs that have been either reported in the curated RNA viral database (Detected) or uniquely detected in the studied soils (Novel) with taxonomic assignments (Phylum) and the known host ranges. Based on the taxonomic ranges of the known hosts, the detected viruses are generally classified into RNA viruses infecting prokaryotes (bar colored in blue) and eukaryotes (bar colored in green) with the rest as unclassified (bar colored in purple). (b) Composition of the RNA viral clusters based on the types of known hosts. A donut chart shows the percentage of the prokaryotic, eukaryotic, and unclassified RNA viral clusters that accounted for the total 867 RNA viral clusters. (c) Taxonomic compositions of the RNA viral clusters. A donut chart demonstrates the relative counts of the RNA viral clusters that are assigned to the taxonomic rank of the Family. The RNA viral families that contain less than 2% of the total RNA viral clusters were combined and shown as the minor group.

A larger fraction of the “classified” RNA viral contigs (those with taxonomic assignment) were attributed to eukaryotic RNA viruses relative to prokaryotic RNA viruses. A total of 1,854 quality-filtered RNA viral contigs were identified from the studied grassland soils under different environmental conditions [i.e., different water content, depth increment, planted/bare, and cultivar type (Table S2)]. Nearly one-third of the RNA viral contigs were novel as they have not been documented in the curated RNA viral reference database (Fig. 2a). The classified RNA viruses spanned a range of phyla including Duplornaviricota, Kitrinoviricota, Lenarviricota, and Pisuviricota. Eukaryotic RNA viruses accounted for 48.3% of the total detected RNA viruses and 74.7% of classified RNA viruses. Because RNA viruses often contain segmented genomes (27), the method of directly clustering RNA viral contigs may overestimate the overall RNA viral diversity. Therefore, the RNA viral contigs were grouped into clusters based on the similarity of their RdRP protein sequences (99% of identity). A similar fraction of eukaryotic RNA viruses relative to prokaryotic RNA viruses was observed whether RNA viruses were considered at the cluster level (47.6% and 11.1%, respectively; Fig. 2b) or contig level (48.3% and 16.3%, respectively; Fig. 2a). The majority of the classified RNA viral clusters were assigned to *Mitoviridae* (31.7% of the total RNA viral clusters or 54.0% of the classified RNA viral clusters), with fungi as the natural hosts (Fig. 2c).

The classified eukaryotic RNA viruses are known to infect a wide variety of eukaryotic members in the soil such as Discoba (excavate protists), Opisthokonta (Fungi/Metazoa group), Rhodophyta (red algae), SAR (a supergroup including Stramenopila, Alveolata, and Rhizaria), and Viridiplantae (green plants) (Fig. 2a). Opisthosomata, containing Fungi and Metazoa with flagellate cells, were identified as the most common hosts for the classified eukaryotic RNA viruses. Prokaryotic RNA viruses were primarily composed of viruses belonging to Leviviricetes of Lenarviricota (Fig. 2a). Viruses within the tentative family *f.0030* of Pisuviricota that was recently re-classified as bacteriophage (7) were detected and accounted as the second most dominant prokaryotic RNA viruses followed by the members belonging to Duplornaviricota.

### Complex RNA viral communities shaped by different environmental factors

To assess the relative complexity between the detected communities, we compared the number of principal components that explained the cumulative proportion of variance within each type of community assessed across samples—the total RNA viral community, eukaryotic community, eukaryotic RNA viral community, prokaryotic community, and prokaryotic RNA viral community. Only two principal components were needed to explain over 95% of variances in SSU-informed eukaryotic and prokaryotic communities under different environmental conditions (Fig. S1). The RdRP-informed RNA viral communities (i.e., the total, eukaryotic, and prokaryotic RNA viral communities), however, displayed a much higher variability under different environmental conditions. To explain over 50% of the cumulative variances, five principal components were needed for prokaryotic RNA viral communities with seven principal components for eukaryotic or total RNA viral communities (Fig. S1). Although different phylogenetic markers may resolve the community composition at different taxonomic levels (or equivalent), the difference in cumulative community variance explained in the function of the number of principal components may also reflect the high complexity in RNA viral communities. The eukaryotic RNA viral community was relatively more complex compared to the prokaryotic RNA viral community of the site (Fig. S1).

**Fig 3:**
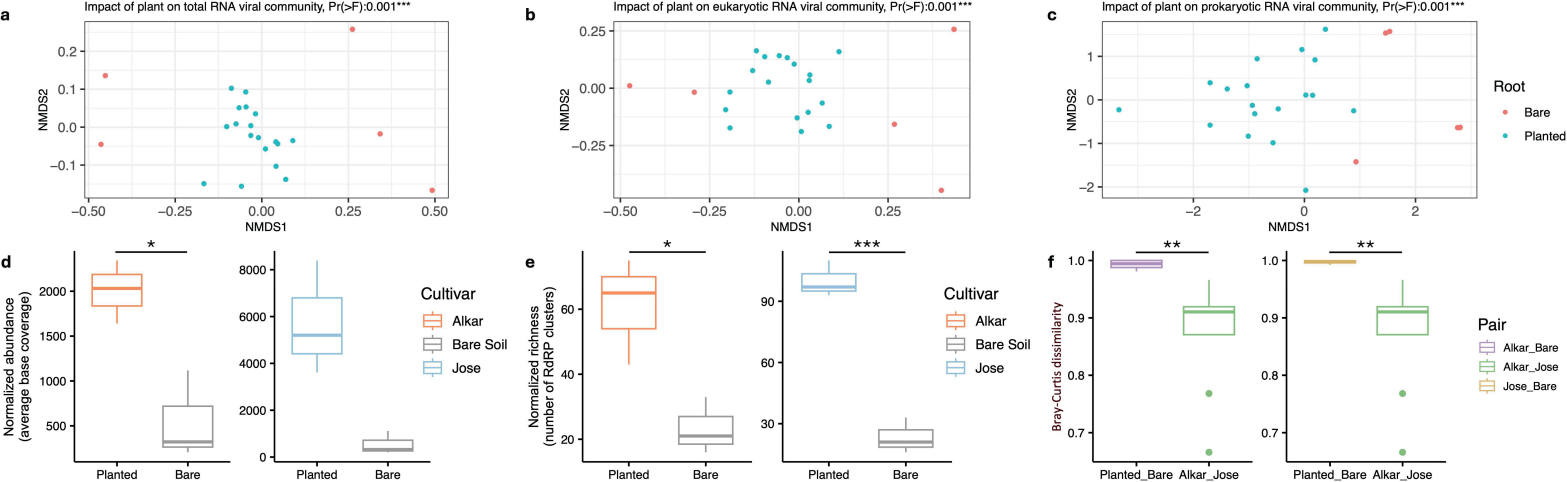
The impact of plant presence/absence on structuring RNA viral communities. Ordination of the total RNA viral communities, eukaryotic RNA viral communities, and prokaryotic RNA viral communities recovered from planted (colored in red) or bare soils (colored in light blue) are assessed by NMDS analysis and shown in panels (a, b, and c), respectively. The significance of the environmental factors’ impacts on community composition was assessed by an F-test. The *P*-values are labeled on the top of each panel. The normalized RNA viral abundance and richness detected in soils planted with either Alkar (colored in orange) or Jose (colored in blue) were compared to the paired bare soils (colored in gray) with the same irrigation intensity (25% WHC) and depth profile (0–5 cm) as shown in panels (d and e). The Bray-Curtis dissimilarities between the total RNA viral communities detected in planted and bare soil were shown in panel (f). The top and bottom of each box represent the 25th and 75th percentiles, and the center line indicates the median. The upper and lower whiskers of each box represent the maximum and minimum values detected in the three biological replicates, respectively. The differences between the compared groups were assessed using a two-sided *t*-test. The significant difference is highlighted by asterisks, with *P* < 0.05 as * and *P* < 0.001 as ***.

**Fig 4:**
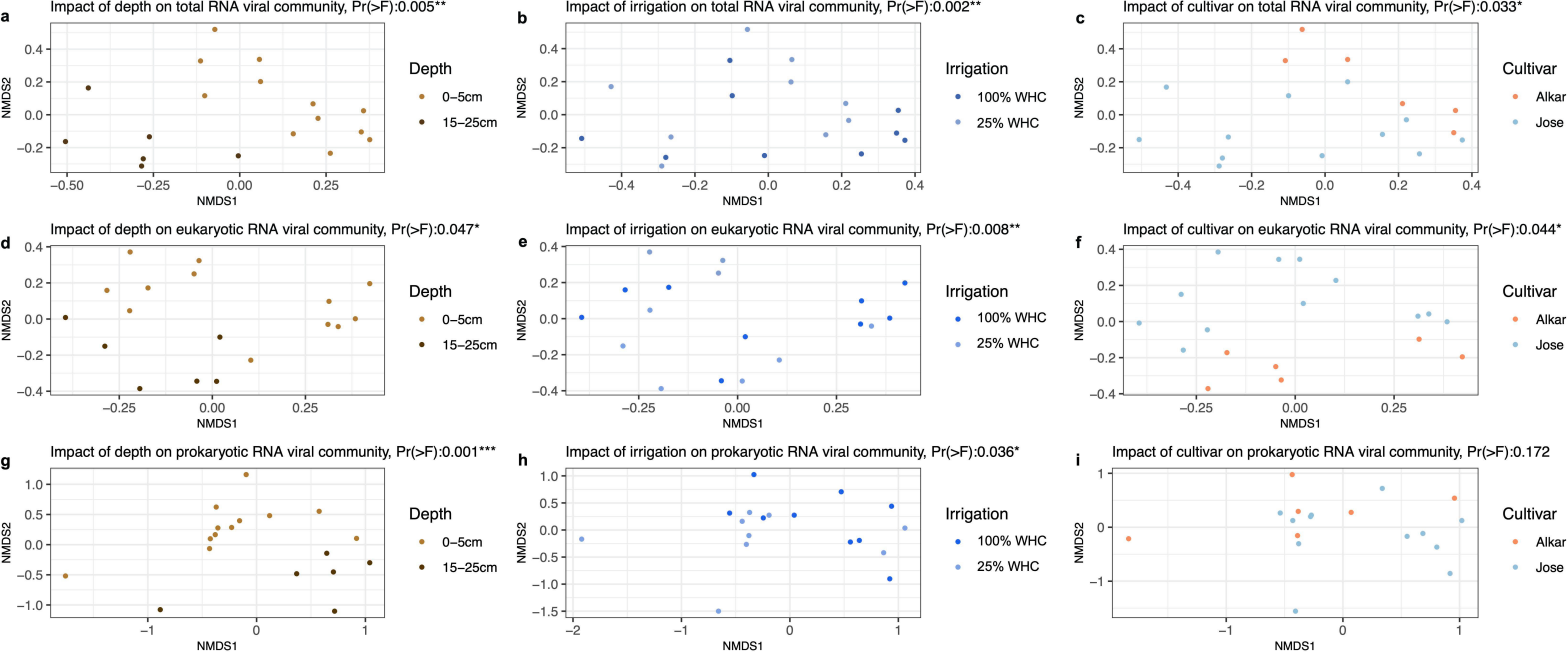
The impact of soil depth, irrigation intensity, and cultivar type on structuring RNA viral communities. The dissimilarities within the RNA viral communities in response to different environmental conditions were assessed by NMDS with an F-test. Ordination of the total RNA viral communities, eukaryotic RNA viral communities, and prokaryotic RNA viral communities recovered from surface soils (0–5 cm, colored in light brown) or deep soils (15–25 cm, colored in dark brown) are shown in panels (a, b, and c), respectively. Ordination of the total RNA viral communities, eukaryotic RNA viral communities, and prokaryotic RNA viral communities recovered from soils with 100% water holding capacity (colored in dark blue) or soils with 25% water holding capacity (colored in light blue) are shown in panels (d, e, and f), respectively. Ordination of the total RNA viral communities, eukaryotic RNA viral communities, and prokaryotic RNA viral communities recovered from soils planted with Alkar (colored in orange) or soils planted with Jose (colored in blue) are shown in panels (g, h, and i), respectively.

We found that the four environmental conditions including water content, plant presence, cultivar, and soil depth significantly impact the soil RNA viral communities (*P* < 0.05, assessed by NMDS analysis). The total, eukaryotic, and prokaryotic RNA viral assemblages were all significantly different between the bulk soils with and without plants (“Planted” versus “Bare” in Fig. 3a through c, *P* < 0.01). The abundance of RNA viral communities in planted soils (“Alkar” or “Jose”) was more than 2.6-fold higher than that in bare soils (Fig. 3d, “Alkar” versus “Bare,” *P* < 0.05). The richness of the RNA viral communities in soil planted with Alkar or Jose was about 1.6- or 3.2-fold higher compared to the RNA viral communities in bare soils (Fig. 3e, “Alkar” versus “Bare,” *P* < 0.05; “Jose” versus “Bare,” *P* < 0.001). The plant cultivars (Alkar or Jose) significantly changed the overall RNA viral community composition of the sites as the dissimilarity of the RNA viral communities between planted and bare soils was higher than that of the RNA viral communities between soils planted with Alkar and Jose (*P* < 0. 01, Fig. 3f). Soil depth, irrigation intensity, and plant cultivar were found to significantly impact the composition of total RNA- and eukaryotic-RNA viral assemblages (Fig. 4a through f; Fig. 1b and S2a). The prokaryotic RNA viruses, however, only responded to the differences in soil depth and irrigation intensity but not to cultivar type (Fig. 4g through i; Fig. S2c).

### Relationships between environmental and community factors

**Fig 5:**
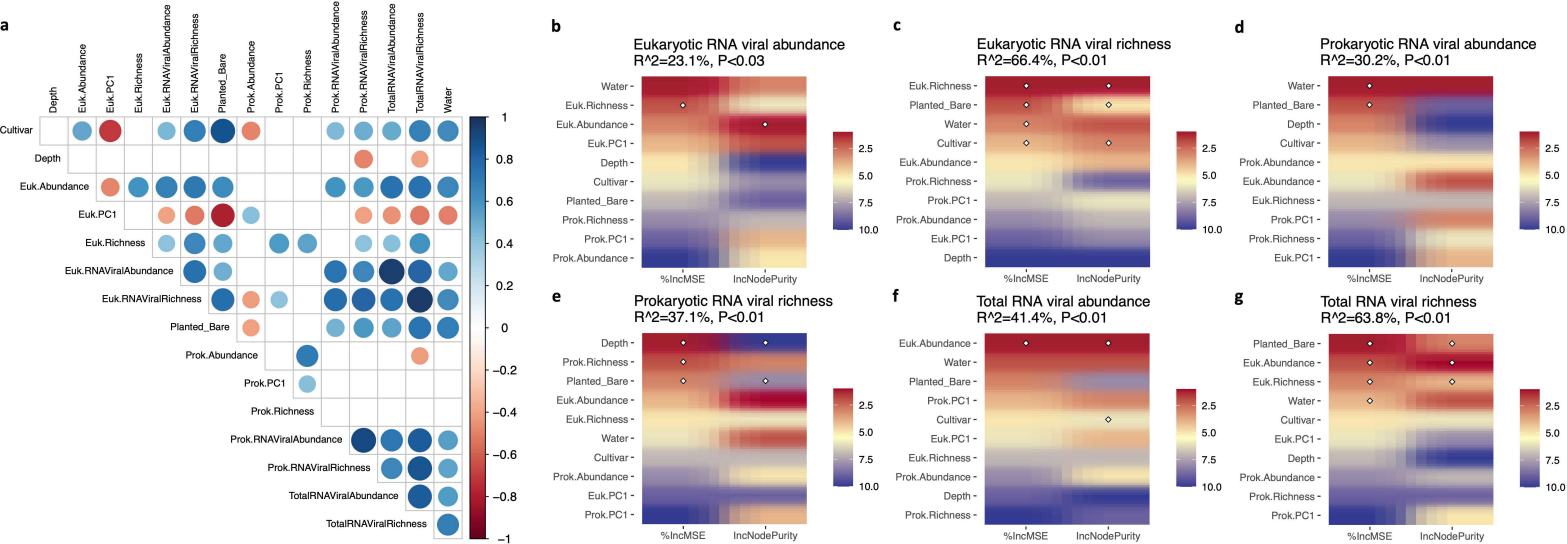
Relationship between environmental and community factors detected in the studied grassland soils. (**a**) Pairwise correlation analysis between the detected environmental and community factors. The community factors include abundance, richness (index for alpha diversity), and the first principal component (PC1, index for beta diversity) of the total RNA viral community (TotalRNA), eukaryotic RNA viral community (Euk.RNA), and prokaryotic RNA viral community (Prok.RNA) as well as the co-existing prokaryotic (Prok) and eukaryotic communities (Euk). The environmental factors include soil depth (Depth), presence of plant (Planted_Bare), type of cultivar (Cultivar), and soil water content (Water). The significant positive and negative correlations (*P* < 0.05) are labeled with blue and red circles. The color gradient and the size of the circles denote the values of the correlation coefficient. (**b–g**) Random forest models show the main environmental and community factors influencing the eukaryotic RNA viral abundance and richness in panels b and c, the prokaryotic RNA viral abundance and richness in panels d and e, and the total RNA viral abundance and richness in panels f and g. The significance of each factor is evaluated by the percentage of the increase in the mean square error (%IncMSE) and increase in node purity (IncNodePurity). The factors that are considered significant (*P* < 0.05) are highlighted with a diamond.

To gain a more mechanistic understanding of how the studied RNA viral communities were assembled, we applied a correlation analysis on all the measured/calculated environmental and community factors and the random forest method to rank the relative importance of factors influencing the RNA viral abundance and richness. The factors with significant correlations are shown in Fig. 5a (Pearson correlation, *P* < 0.05). Unlike soil depth, the presence of plants (Planted_Bare), cultivar types (Cultivar), and soil water content (Water) showed significant associations with prokaryotic and/or eukaryotic communities in addition to all RNA viral communities (Fig. 5). We hypothesized that these factors may impact RNA viral communities by influencing the viral host populations (mostly eukaryotes). This hypothesis was supported by the strong associations between eukaryotic communities and eukaryotic RNA viral communities (Fig. 5a through c). In contrast, the total and prokaryotic RNA viral communities did not show a strong relationship with the detected prokaryotic communities (Fig. 5a, d, and e). This may be explained by the higher proportion of eukaryotic RNA viruses within the classified RNA viral communities (Fig. 2).

**Fig 6:**
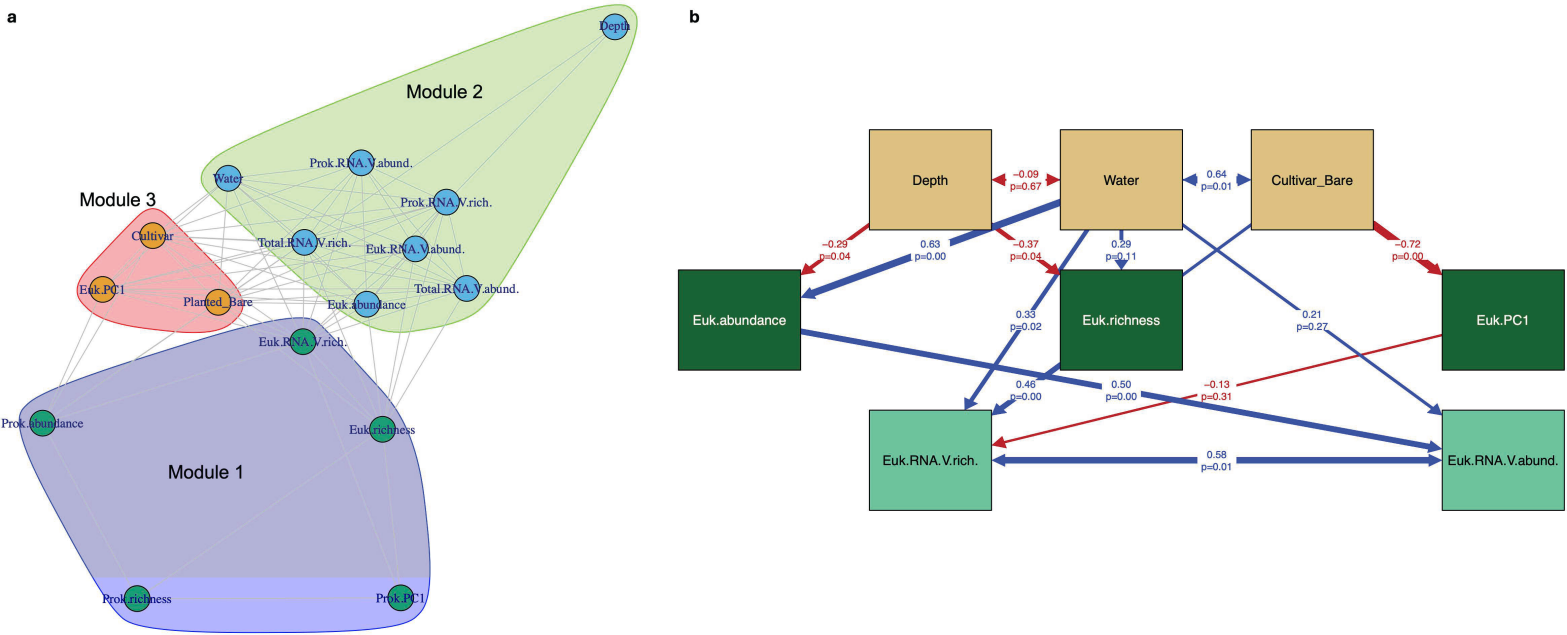
Potential interactions between the environmental and community factors that influence soil RNA viral communities. (**a**) A network showing the clustering of the environmental and community factors. The nodes represent only the significant factors identified in the correlation analysis and random forest models. The nodes with the same color are clustered into the same module delineated by factors that were more connected within modules but less linked with the factors in other modules. The three identified modules are colored red, green, and purple, respectively. (**b**) SEM was applied to test the relationships between environmental properties (brown boxes), eukaryotic communities (dark green boxes), and eukaryotic RNA viral communities (light green boxes). The environmental factors include soil depth (Depth), soil water content (Water), presence of plant, and type of cultivar (Cultivar_Bare). The eukaryotic abundance (Euk.abundance), richness (index for alpha diversity, Euk.richness), and the first principal component (index for beta diversity, Euk.PC1) are used to represent the co-existing eukaryotic community. Euk.RNA.V.rich. and Euk.RNA.V.abund. represent eukaryotic RNA viral richness and abundance, respectively. Parameters evaluating the model fitness were Chi-square (χ2) = 18.17, *P* = 0.15, and comparative fit index, or CFI = 0.94. The direction of the arrows represents the direct impact of one variable on another supported by SEM. Blue and red arrows represent positive and negative pathways, respectively. Arrow width is proportional to the strength of the relationship, and the numbers on the arrows are the path coefficients and the *P*-value.

The significant associations identified by the correlation matrix and random forest method were converted into a factor network for modularity analysis to inform the high-level structures between environmental and community factors. The factor network is structured into three modules with each containing factors that were more connected to each other but less connected to the factors in other modules (optimized modularity score = 0.08, Fig. 6a). Community factors describing the eukaryotic and prokaryotic communities (i.e., richness, abundance, or PC1) were generally grouped into one module (nodes colored in green, area colored in purple, Module 1) except for the abundance estimate of the eukaryotes (Euk.abundance). Eukaryotic abundance was more connected to the factors describing the RNA viral communities in Module 2 (nodes colored in blue, area colored in light green) along with soil water content and sampling depth (“Water” and “Depth”), indicating that these environmental factors were more strongly associated with community factors of the detected RNA viruses than cultivar type (Cultivar) and plant presence (“Planted_Bare” in Module 3 with nodes colored in orange and area colored in red). The results of module optimization provided a correlation-based framework to build the structural equation model for further investigations on the directional causal relationships among the factors as described below.

### Drivers of eukaryotic RNA viral community assemblages

We further investigated the main drivers only for the eukaryotic RNA viral community assemblages because the detected RNA viruses were mainly eukaryotic viruses and the prokaryotic communities showed fewer connections with the RNA viral communities (Fig. 6). Based on the framework indicated by the network modules, an SEM was constructed in three layers, environmental factors (i.e., “Depth,” “Water,” and “Cultivar_Bare”), eukaryotic communities (i.e., “Euk.abundance,” “Euk.richness,” and “Euk.PC1”) and eukaryotic RNA viral assemblages (i.e., “Euk.RNA.V.rich” and “Euk.RNA.V.abund”) (Fig. 6b). The directional interactions between factors across layers were optimized to provide a good model fit as evaluated by a Chi-squared (χ2) test (χ2 = 18.17, *P* = 0.15) and comparative fit index (CFI = 0.94). The SEM is intended to inform potential causal relationships between the examined factors and generate hypotheses to test in future studies.

The SEM analysis indicated that subsurface soils had significantly reduced the abundance and richness of soil eukaryotes and thus indirectly affected the eukaryotic RNA viral assemblages. Soil water content showed both direct and indirect impact on eukaryotic RNA viruses as higher soil water content was related to higher eukaryotic viral abundance and richness as well as a positive influence on eukaryotic communities. For simplicity, we transformed the type of cultivars and the presence of plants into numerical values and collectively named it “Cultivar_Bare” with “Jose,” “Alkar,” and “Bare” soil as 2, 1, and 0, respectively. “Cultivar_Bare” was found to significantly influence the β-diversity of the eukaryotic community represented by PC1 from principal component analysis and indirectly impact eukaryotic RNA viral richness. The richness and abundance of soil eukaryotes were predicted to have significantly positive impacts on the richness and abundance of soil RNA viruses, respectively.

## DISCUSSION

Studies on soil RNA viral communities are still in their infancy. A consensus has not yet been reached on the relative dominance of eukaryotic RNA viruses versus prokaryotic RNA viruses in soil (estimated by the number of unique contigs/vOTUs). In our study conducted in arid grassland soils from a field experiment representing multiple environmental and management treatments, we found that the normalized abundance of eukaryotic RNA viruses was nearly three times higher than that of prokaryotic RNA viruses. This observation aligns with the few studies available to date on RNA viral communities in soils (12, 13, 16). In a California annual grassland, the abundance of eukaryotic RNA viral contigs was about 1.8 times greater than the prokaryotic RNA viral contigs (12). A higher normalized relative abundance of eukaryotic RNA viruses compared to prokaryotic RNA viruses was also reported in a Kansas grassland (16) and thawed permafrost (13). The dominance of RNA viruses that infect eukaryotic hosts rather than prokaryotic hosts may be generalizable to other soils and/or ecosystems. A recent global RNA viral study demonstrated that prokaryotic RNA viruses accounted for less than 25% of the RNA viruses detected in the metatranscriptomic data sets sequenced on the total RNA (7). Together, these emerging results point to the predominance of eukaryotic RNA viruses in soil RNA viral communities, though more studies are needed to evaluate this trend more broadly. Furthermore, new protocols targeting double-stranded RNA viruses are needed to complement the current method and provide more comprehensive profiling of the complex soil RNA viral communities. We acknowledge the historical bias toward animal viruses and the resulting possibility of misannotated viral hosts in the public databases used for viral identification. Thus, we encourage more community efforts in validating and updating virus-host databases to improve the accuracy of viral host assignments enabling cross-study comparison.

The methodological approach used to evaluate RNA viruses influences which subset of the total RNA viral community is being considered. Here, we analyzed metatranscriptomes sequenced from total soil RNA, enabling the detection of RNA viruses that transmit through an extracellular phase as well as those that remain intracellular, which is complementary to the approach that sequences RNA following enrichment of only extracellular virus-like particles (10). Diverse RNA viral communities spanning four of the five International Committee on Taxonomy of Viruses-classified RNA viral phyla (which use RdRP to replicate) (28) were detected in the studied grassland soils. In these bulk metatranscriptomic data, sequences were recovered from RNA viruses such as *Gammapartitivirus* belonging to *Partitiviridae* that infect ascomycetous fungi and are only transmitted during cell division/fusion (29). The approach implemented in this and other studies, however, relies on using RdRP sequences for viral taxonomic identification and therefore underestimates the contribution of eukaryote-infecting *Retroviridae* that replicate without using RdRP (30). Metatranscriptomes generated without rRNA depletion or polyA enrichment (known as a metatranscriptome sequenced on total RNA) enable SSU rRNA-based profiling of the co-existing prokaryotic and eukaryotic communities that contain potential hosts for the detected RNA viruses*.* We leveraged this advantage to also analyze co-existing eukaryotic and prokaryotic communities and found that the variation in the RNA viral communities recovered in soil under different environmental conditions was much higher compared to that in the prokaryotic and eukaryotic communities from the same samples. Viral replication is highly dependent on host activity and metabolic machinery (31). Therefore, the variation observed within the RNA viral community in response to environmental perturbations is suspected to be similar to that of their hosts. However, we found a much higher dissimilarity in RNA viruses across samples under different environmental conditions compared to their hosts. This result suggests that factors beyond the prokaryotic and eukaryotic communities may regulate the composition of RNA viral communities in soil. We then further examine some of these factors measured/calculated in this study.

The design of our field experiment, which has been ongoing for 5 years, provided an opportunity to concurrently evaluate the contributions of multiple environmental factors to shaping RNA viral communities at a field scale. These factors include water content, presence of plant, type of plant cultivar, and soil depth which may directly or indirectly (through effects on host communities) impact soil RNA viral communities. Although the metatranscriptomes were sequenced from bulk soils, rather than rhizosphere (root-associated) soils, the presence of plants (i.e., bare soils versus planted soils) plays a strong role in structuring the soil RNA viral communities. This is because plant cover promotes biodiversity in soil (32) and may also support the growth of the eukaryotic members, in turn supporting the associated RNA viruses. After removing the bare soil samples from the ordination analysis, the significant impact of soil depth increment, irrigation intensity, and type of cultivar on the RNA viral communities emerged. We hypothesize that these factors directly influence soil viruses (e.g., the fate of RNA viruses in soils) as well as indirectly influence the RNA viral community composition by affecting the prokaryotic and eukaryotic communities. The factor network structure supports the hypothesis of the direct and indirect impacts of environmental factors on soil RNA viral communities. The direct effects hypothesis is supported by the modularity analysis on the factor network that shows environmental factors such as soil water content (Water) and depth (Depth) were directly clustered with the factors representing RNA viral communities. In contrast, the environmental factors such as type of cultivar (Cultivar) and presence of plant (Planted_Bare) were indirectly linked to the module with the RNA viral community factors. This indirect link is suspected to be a directional impact from the environmental factors through eukaryotes and prokaryotes that include hosts of the soil RNA viruses and then to the detected RNA viruses.

The hierarchical structure (i.e., the direct and indirect effects on RNA viral communities) of environmental and community factors was supported by structural equation modeling, at least for eukaryotic viral communities. The SEM provides a better model fit for eukaryotic and eukaryotic RNA viral data than prokaryotic and prokaryotic RNA viral data. This could be due to the current understanding that the most abundant eukaryotes (i.e., plants and fungi) in soil are associated with RNA viruses (13) and the majority of the dsDNA viruses detected from bulk soil metagenomes and viromes are bacteriophages (33). The water content (Water) shows a strong positive impact directly on eukaryotic RNA viral richness (“Water” to “Euk.RNA.V.richness”) and indirectly on eukaryotic RNA viral abundance (“Water” to “Euk.abundance” to “Euk.RNA.V.abund”). Increasing soil water content, especially in sandy soils as studied here, is expected to improve viral transport in the soil matrix increasing the spatial turnover rate of the viral communities and thus a higher viral richness per unit of soil sampled (34). The indirect impact of water content on eukaryotic RNA viruses may be a result of higher soil water content alleviating water stress for viral hosts in this semi-arid soil and/or promoting nutrient mobility to support the growth (abundance) of the hosts and associated viruses. Plant presence (Cultivar_Bare) also had a direct impact on the richness of eukaryotic RNA viruses. In addition to serving as hot spots of inter-species interactions (35), plant roots may serve as the media to retain RNA viruses. Viral adsorption to particulate materials has been reported to improve the survival of RNA viruses in terrestrial and aquatic systems (34). This may explain why the relative abundance of the dominant eukaryotic RNA viruses belonging to *Mitoviridae* was less abundant in bare soils in contrast to the planted soils, while their host, fungal communities, did not follow the same pattern (Fig. S3 and S4). Our previous study has shown the different ecotypes of tall wheatgrass recruit distinct microbiomes (24). Similarly, different RNA viral communities were recovered in soils planted with Alkar and Jose. Alkar is a native cultivar bred in the northwest of the North American Continent (e.g., the studied grassland) and Jose is introduced from the southwest USA because of its tolerance to saline and alkaline soils (36). The native cultivar is suspected to retain eukaryotic and prokaryotic communities with members that may gain resistance to viral infections after the co-evolution with viruses of the site. This may explain why less abundant and less diverse RNA viruses were detected in the soils with Alkar than in soils with Jose. Although soil “Depth” was clustered with the factors representing RNA viral communities in the network analysis, the SEM analysis suggests a strong negative relationship with eukaryotic communities. This negative relationship is consistent with the previous report that eukaryotes were mainly detected in the upper soil layer and their richness decreased with depth (37). The SEM analysis provides a conceptual framework of how the environmental factors shape the eukaryotic RNA viral communities in soil and can be further tested in other studies.

Our findings collectively underscore the possible predominance of eukaryotic RNA viruses within these grassland RNA viral communities. They also emphasize the need for further research and the development of new protocols aimed at understanding the ecological significance of double-stranded RNA viruses. We recovered novel RNA viral communities from metatranscriptomes sequenced from grassland soils that have been exposed to a range of field treatments. The classified RNA viral communities are dominated by eukaryotic RNA viruses belonging to *Mitoviridae*. Our statistical analyses suggest that environmental factors have both direct and indirect impacts on soil RNA viral communities. For example, soil water content, presence of plant, and type of cultivar may have a direct positive impact on eukaryotic RNA viral richness and an indirect impact on eukaryotic RNA viral abundance via influencing the co-existing eukaryotic species. This study proposes an integrated framework to study the complex interactions between environment and microbial communities in the field and inform their potential causal relationship that can be tested in incubation studies.

## Data Availability

The 24 metatranscriptome data sets and the metadata of each soil sample are deposited in DataHub (https://data.pnnl.gov/group/nodes/dataset/33363, DOI: https://doi.org/10.25584/1986545, the sequencing data used in this study is specified in Table S1. The identified RNA viral contigs are deposited and publicly available at DataHub (https://data.pnnl.gov/group/nodes/dataset/33706). The curated RNA viral reference database is available at Zenodo (https://doi.org/10.5281/zenodo.10989253). The R codes used for the statistical analyses and figure plotting are available at https://github.com/Ruonan0101/RNA_viral_communties_environmental_perturbations.

## SUPPLEMENTAL MATERIAL

Supplemental figures msystems.00099-24-s0001.docx Figures S1 to S4.

Supplemental tables msystems.00099-24-s0002.xlsx Table S1 and S2.

ASM does not own the copyrights to Supplemental Material that may be linked to, or accessed through, an article. The authors have granted ASM a non-exclusive, world-wide license to publish the Supplemental Material files. Please contact the corresponding author directly for reuse.
