# Supplementary material for: The direct and indirect drivers shaping RNA viral communities in grassland soils
Source: mSystems. 2024 Jul 9;9(8):e00099-24. doi: 10.1128/msystems.00099-24 (PMC11334463; doi:10.1128/msystems.00099-24)
Supplement: Supplemental figures — Figures S1 to S4. [file msystems.00099-24-s0001.docx]

**Supplementary figures**

**The direct and indirect drivers shaping RNA viral communities in grassland soil**

Ruonan Wu^1^, Amy E. Zimmerman^1^, Kirsten S. Hofmockel^1,2#^

^1^Earth and Biological Sciences Directorate, Pacific Northwest National Lab, Richland, Washington, USA

^2^Department of Agronomy, Iowa State University, Ames, Iowa, USA

#Address correspondence to Kirsten S Hofmockel, kirsten.hofmockel@pnnl.gov.

**Figure S1 The relative complexity of the eukaryotic, prokaryotic, and RNA viral communities distributed across sites.** The relative complexity of the detected communities is compared by the number of the principal components (x-axis) needed to explain the cumulative proportions of the total variance observed for each type of community across sites (y-axis). The values of the data points were retrieved from the principal component analysis of each community type across the 24 samples.

**Figure S2 The impact of soil depth, irrigation intensity, and cultivar type on structuring the total, eukaryotic and prokaryotic RNA viral communities.** The dissimilarities within the RNA viral communities in response to different environmental conditions were assessed by NMDS with an F-test. Ordination of the total RNA viral communities, eukaryotic RNA viral communities, and prokaryotic RNA viral communities are shown in panels a, b and c, respectively. The viral communities recovered from surface soils (0-5 cm) or deep soils (15-25 cm) are labeled in circles and triangles, respectively. Soils planted with Alkar and Jose were labeled in orange and blue, respectively. The size of the data points differentiates the soils with different irrigation treatments, i.e., 100% water holding capacity colored in dark blue and 25% water holding capacity colored in light blue.

**Figure S3 The community composition of the eukaryotic populations that contain known hosts of the detected RNA viruses.** The heatmap illustrates abundance estimates for each eukaryotic taxon that was identified as the host taxon of the detected RNA viruses. The estimated abundances were log-transformed, and warmer colors represent higher abundances. The abundance profile was grouped according to the similarity of the community composition.

**Figure S4 RNA viral community composition of the site**. The heatmap illustrates abundance estimates for each eukaryotic RNA viral taxon detected in the 24 soil samples. The estimated abundances were log-transformed, and warmer colors represent higher abundances. The abundance profile was grouped according to the similarity of the community composition.
